# Clinical Value of Serum miRNA in Patients with Acute Promyelocytic Leukemia

**DOI:** 10.1155/2022/7315879

**Published:** 2022-04-01

**Authors:** Bei Zhang, Zhixin Pei, Hongxia Wang, Junjun Bai, Junjie Wang, Yingxin Zhao, Mei Jiang, Huimin Wu, Qinglin Song

**Affiliations:** ^1^Department of Hematology, Jiaozuo People's Hospital, Jiaozuo 454000, China; ^2^Department of Nuclear Medicine, The Second Affiliated Hospital of Henan Polytechnic University, Jiaozuo 454000, China

## Abstract

**Objective:**

To explore the clinical value of specific miRNA in patients with acute promyelocytic leukemia.

**Methods:**

129 patients with acute promyelocytic leukemia diagnosed in our hospital from January 2015 to January 2020 were selected as the observation group. At the same time, 74 patients with nonacute promyelocytic leukemia who underwent bone marrow aspiration were included as the control group. The expression levels of miR-126-5p and miR-13, different characteristic parameters, and prognosis were compared between the two groups, and the clinical significance of miR-126-5p and miR-13 in acute promyelocytic leukemia was analyzed.

**Results:**

The expression of miR-126-5p (12.31 ± 2.25 versus 17.30 ± 3.28) and miR-13 (16.05 ± 3.47 versus 21.66 ± 2.18) in the observation group was significantly lower than that in the control group (*P* < 0.05). The expression level of miR-126-5p was significantly correlated with lactate dehydrogenase level, HGB level, NPM1 mutant type, and complete remission (*P* < 0.05). The expression level of miR-13 was significantly correlated with HGB level, NPM1 mutant type, and complete remission (*P* < 0.05). Both expression levels of miR-126-5p and miR-13 were not correlated with sex, age, WBC, PLT, proportion of bone marrow primordial cells, hepatomegaly, splenomegaly, lymph node enlargement, and FLT3-ITD (*P* > 0.05). Cox multivariate regression analysis showed that peripheral blood WBC, bone marrow blast cell count, and miR-126-5p and miR-13 were prognostic factors in patients with acute promyelocytic leukemia (*P* < 0.05). The sensitivity, specificity, accuracy, and AUC of serum miR-126-5p prediction were 75.83%, 84.56%, 82.17%, and 0.729, respectively. The sensitivity, specificity, accuracy, and AUC of serum miR-13 prediction were 78.64%, 88.49%, 86.20% and 0.882, respectively.

**Conclusion:**

Serum miR-126-5p and miR-13 are closely related to the prognosis of patients with acute promyelocytic leukemia. Serum miR-126-5p and miR-13 can be used as reliable indexes to predict the prognosis of patients.

## 1. Introduction

From a historical point of view, the genome contains coding regions and noncoding regions, of which noncoding regions were once considered to be nonfunctional [1]. However, many studies have shown that these noncoding regions are involved in a variety of cell growth, proliferation, and differentiation, among which miRNA plays an important role [2, 3]. Although the length of miRNA sequence is very short, it participates in almost all intracellular signal pathways. The sensitive regulation of these noncoding small RNAs is an important factor in maintaining intracellular homeostasis. Therefore, the study of miRNA is of great significance for exploring the physiological and pathological phenomena of diseases [4].

miRNA is a class of naturally occurring short noncoding RNA molecules [5, 6]. miRNA account for 1-5% of the human genome [7]. Recent studies have shown that the human gene of 10%-20% is the target gene regulated by miRNA [8]. miRNA plays an important role in the pathogenesis of many diseases, including malignant hematological diseases. More and more evidence shows that abnormal expression of miRNA is common in malignant hematological diseases [9]. In recent years, it has been found that miRNA is related to the development of malignant hematological diseases [10].

miRNA plays an important role in the pathogenesis and cell differentiation of acute myeloid leukemia (AML) [11]. The AML is characterized by cytogenetic changes or mutations, most of which are located in transcription factors and a variety of tumor suppressor genes [12, 13]. In recent years, miRNA has been identified as a new mechanism of gene regulation. miRNA affects cell differentiation, growth, and apoptosis by pairing with target mRNA and participates in the pathogenesis of leukemia [14]. The level of miRNA expression in normal and leukemic cells is different, and different leukemic subtypes can be distinguished [15]. Because the mutation of miRNA is rare, the variation of miRNA expression level is the main factor controlling the effect of miRNA [16]. These small single-stranded RNAs were initially thought to regulate protein translation and have been confirmed to affect mRNA stability and induce mRNA degradation [17]. In [18], miR-26a/b and miR-29b are upregulated during osteogenic differentiation of USSC and share target genes inhibiting osteogenesis. Garzon et al. [19] studied the leukemic cells of newly diagnosed acute promyelocytic leukemia (APL) patients taking all-transretinoic acid by quantitative RT-PCR (qRT-PCR). The results showed that miR-16-1, let-7-7a, let-7c, let-7d, miR-223, miR-342, and miR-107 were upregulated, while miR-181b was downregulated. The expression of miRNA has the potential to distinguish different subtypes in the diagnosis of ALL, in order to understand the pathogenesis and differential diagnosis and treatment of leukemia. Some studies have found that miR-128a and miR-128b are overexpressed in ALL, while let-7b and miR-223 are downregulated. According to this, ALL and AML can be distinguished, and the diagnostic accuracy is >95% [20].

In order to explore the relationship between the expression characteristics of miRNA in chronic lymphoblastic leukemia (CLL) and known prognostic factors, Crespo et al. studied 56 patients with CLL and found that there was little or no mutation in IgVH gene, that is, those with high expression of ZAP-70 protein had high invasiveness and low survival rate [21]. In CLL, a unique level of miRNA expression is associated with prognostic factors and disease progression. miRNA transcriptional mutations occur frequently, and miRNA has an important function [22]. Cimmino et al. [23] further studied the pathway of miRNA-15 and miR-16 and found that both of them can inhibit the apoptosis of B lymphoma cells by downregulating the expression of bcl-2 protein. miRNA plays an important role in cell proliferation, differentiation, and apoptosis by regulating the transcription of target genes and plays an important role in tumorigenesis. Some miRNA may participate in the form of tumor by regulating cell proliferation and apoptosis, while others may regulate tumorigenesis through target oncogenes or tumor suppressor genes. The study of the expression of miRNA in malignant hematological diseases by different detection methods can promote the further understanding of the pathogenesis of leukemia and malignant lymphoma and is an important index for diagnosis, treatment, and prognosis judgment [24]. The purpose of this study was to analyze the expression of miRNA-126-5p and miRNA-13 in acute promyelocytic leukemia and their correlation with clinicopathological features, in order to explore the expression of miR296 and miR517c in acute promyelocytic leukemia and its value in predicting the prognosis.

## 2. Materials and Methods

### 2.1. Study Design and Participants

129 patients with acute promyelocytic leukemia diagnosed in Jiaozuo People's Hospital and The First Affiliated Hospital of Zhengzhou University from January 2015 to January 2020 were selected as the observation group. At the same time, 74 patients with nonacute promyelocytic leukemia (acute leukemia except acute promyelocytic leukemia) who underwent bone marrow aspiration were included in the control group. There were 59 males and 70 females in the observation group, with an average age of 37.1 ± 4.5 years old, BMI: 21.89 ± 1.69 kg/m^2^, and 36 males and 38 females in the control group, with an average age of 36.4 ± 5.3 years old. BMI: 21.93 ± 1.26 kg/m^2^. There was no difference in the condition, course, and general data of the selected patients (*P* > 0.05), which was comparable. All the subjects obtained informed consent. The ethics committee of Jiaozuo People's Hospital approved this research plan. All participants underwent a complete medical history examination and clinical examination.

### 2.2. Reagents and Instruments

The miRNA extraction kit, miRNA cDNA first chain synthesis kit, and miRNA fluorescence quantitative PCR detection kit were purchased from Beijing Tiangen Company, RIPA lysate and BCA protein quantitative kits were purchased from Shanghai Biyuntian Company, and the first antibodies of Foxo3a, p21, p27, and Bim were purchased from Abcam Company. The fluorescence quantitative PCR instrument is from ABI Company, and the chemical developer is from Shanghai Tianneng Company.

### 2.3. Serum miRNA-126-5p and miRNA-13 Expressions Were Detected, Genomic DNA Was Extracted, and Gene Mutation Was Detected

Detection of serum miRNA-126-5p and miRNA-13 levels 5 ml of fasting venous blood was collected by using Trizol reagent kit (invitrogen Co., USA, item number 16684-029) to extract total RNA and then quantified and reverse transcribed to obtain cDNA. The PCR reaction system was configured by miRNA fluorescence quantitative PCR detection kit, according to the two-step reaction procedure: 95°C 5 s and 60°C 15 s repeated 40 cycles, miRNA-126-5p, miRNA-13, and U6 were amplified, respectively. According to the cycle curve and cycle threshold, U6 was used as the housekeeper gene to calculate the expression of miRNA-126-5p and miRNA-13. Exon 12 of NPMl gene and FLT3-ITD mutation were detected by genomic DNA-PCR in 129 patients with acute promyelocytic leukemia.

### 2.4. Evaluation of Curative Effect

129 patients were hospitalized for treatment. Patients with high expression level of leukocytes were pretreated with hydroxyurea alone or combined with leukocyte clearance before induction chemotherapy. All patients received two courses of standard dose DA (daunorubicin + cytarabine) or IA (desmethoxy daunorubicin + cytarabine) induction chemotherapy. Patients with complete remission (CR) continued to receive consolidation and maintenance with combined chemotherapy, and 13 of them received hematopoietic stem cell transplantation in the CRl or CR2 phase. Patients without CR were treated with FLAG±IDA (fludarabine + cytarabine + granulocyte colony stimulating factor ± desmethoxydaunorubicin) regimen.

### 2.5. Follow-Up

The total survival time (OS) of each patient was calculated by means of follow-up. The way of follow-up of inpatients is to consult patients' cases, and the way of follow-up of discharged patients is telephone or outpatient follow-up. The time of OS is the time from diagnosis to death or the time of the last follow-up.

### 2.6. Statistical Analysis

This is a retrospective study. The data were statistically processed by SPSS25.0 software, the measurement data were expressed as median, the differences between groups were compared by *t*-test, and the counting data were expressed by frequency and *χ*^2^ test. The receiver working curve (ROC) was used to analyze the predictive efficacy of miRNA-126-5p and miRNA-13 in predicting the risk of acute promyelocytic leukemia. The survival data were described by Kaplan-Meier method, and the survival curves were compared by Log-rank test. Cox risk model was used to analyze the factors affecting the prognosis of patients, and *P* < 0.05 was considered to be statistically significant.

## 3. Result

### 3.1. Comparison of miR-126-5p and miR-13 Expression Levels between the Two Groups

The relative expression of miR-126-5p in observation group was 12.31 ± 2.25, significantly lower than 17.30 ± 3.28 in the control group (*P* < 0.05). The relative expression of miR-13 in the observation group was 16.05 ± 3.47, significantly lower than 21.66 ± 2.18 in the control group (*P* < 0.05) (Table 1).

### 3.2. Comparison of miR-126-5p and miR-13 Expression Levels in Patients with Different Characteristics

The expression level of miR-126-5p was significantly correlated with lactate dehydrogenase level, HGB level, NPM1 mutant type, and complete remission (*P* < 0.05), but not with sex, age, WBC, PLT, proportion of bone marrow primordial cells, hepatomegaly, splenomegaly, lymph node enlargement, and FLT3-ITD (*P* > 0.05). The expression level of miR-13 was significantly correlated with HGB level, NPM1 mutant type, and complete remission (*P* < 0.05), but not with sex, age, lactate dehydrogenase level, WBC, PLT, proportion of bone marrow primordial cells, hepatomegaly, splenomegaly, lymph node enlargement, and FLT3-ITD (*P* > 005).  Table 2.

### 3.3. Predictive Efficacy of miR-126-5p and miR-13 on the Risk of Acute Promyelocytic Leukemia

**Figure 1:**
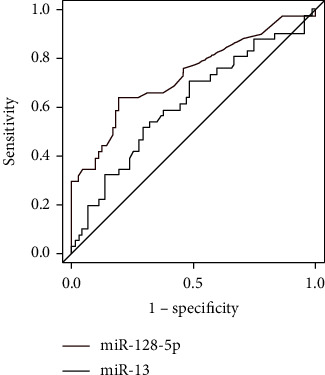
ROC curve of serum miR-126-5p and miR-13 in predicting the prognosis of patients with acute promyelocytic leukemia.

The best cut-off point for the prediction of 1-year death in patients with acute promyelocytic leukemia by analyzing the relative expression of serum miR-126-5p and miR-13 was 5.69, and the sensitivity, specificity, accuracy, and AUC of serum miR-126-5p were 75.83%, 84.56%, 82.17%, and 0.729, respectively. The sensitivity, specificity, accuracy, and AUC of serum miR-13 prediction were 78.64%, 88.49%, 86.20%, and 0.882, respectively (Figure 1).

### 3.4. Effect of miR-126-5p and miR-13 Expression on Prognosis of Patients with Acute Promyelocytic Leukemia

**Figure 2:**
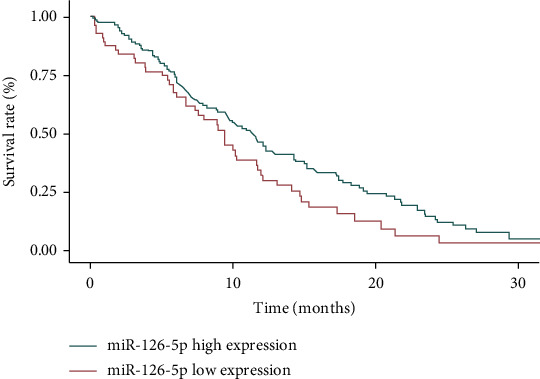
The effect of miR-126-5p expression on the prognosis of acute promyelocytic leukemia.

**Figure 3:**
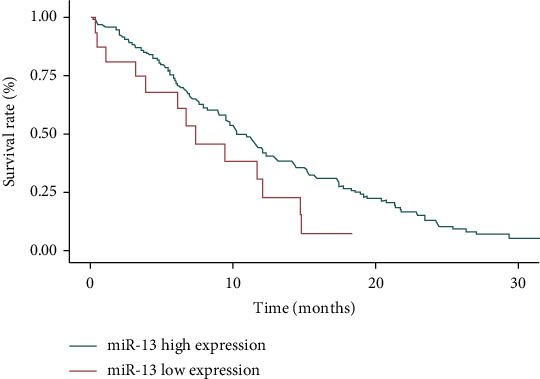
The effect of miR-13 expression on the prognosis of acute promyelocytic leukemia.

The median follow-up time of 129 patients was 19.4 (9-58) months. The median OS time of patients with high miR-126-5P expression was 34 months (95% CI: 13.9-48.1 months). The median OS time of patients with low miR-126-5p expression was 22 months (95% CI: 11.7-36.2 months), and the median OS time of patients with high miR-126-5p expression was significantly higher than that of patients with low miR-126-5P expression (*P* < 0.05, Figure 2). The median OS time of patients with high miR-13 expression was 31 months (95% CI: 12.7-43.5 months). The median OS time of patients with low miR-13 expression was 19 months (95% CI: 10.4-32.9 months), and the median OS time of patients with high miR-13 expression was significantly higher than that of patients with low miR-13 expression (*P* < 0.05, Figure 3).

### 3.5. Multivariate Analysis of Prognostic Factors in Patients with Acute Promyelocytic Leukemia

Cox multivariate regression analysis showed that peripheral blood WBC, bone marrow blast cell count, and miR-126-5p and miR-13 were prognostic factors in patients with acute promyelocytic leukemia (*P* < 0.05) (Table 3).

## 4. Discussion

APL is a kind of AML with abnormal promyelocytic malignant proliferation and recurrent cytogenetic abnormalities [25]. It is a kind of hematological malignant tumor which is dangerous and has recurrent cytogenetic abnormalities. It has been found that miRNAs regulate different stages of cell development of hematological tumors in the form of post-transcriptional regulation by degrading target RNA or inhibiting the translation of target proteins [26]. Previous studies have shown that miRNAs play an important role in APL, they can be used not only as oncogenes but also as tumor suppressor genes, which provides a new marker for the diagnosis and treatment of APL [27]. Recent studies have shown that miRNAs play an important regulatory role in the process of hematopoietic differentiation in mammals [28]. miRNA-181a, miRNA-223, and miRNA-142s have been proved to be specifically expressed in the hematopoietic system miRNAs, dynamically expressed in the early hematopoiesis and lineage differentiation, and aberrant expression of miRNAs can change the hematopoietic lineage differentiation. During the differentiation of myeloid progenitor cells into megakaryocytes, microRNA-22 promotes megakaryocyte differentiation through repression of its target, GFI1 [29].

In recent years, a large number of studies have shown that the cause of leukemia is not only limited to chromosome abnormalities and gene mutations but also closely related to epigenetic and miRNA expression changes [30]. miRNAs not only inhibit the expression of target genes at the post-transcriptional level but also its expression is regulated by epigenetic mechanisms and acts directly or indirectly on key epigenetic enzymes such as DNA methyltransferase (DNMTs) [31]. Some researchers believe that because the abnormal expression of miRNAs is related to the occurrence and development of many diseases, and its molecular weight is small, it is easy to release human blood, and it is very stable and difficult to degrade in plasma and serum, so it can be used as a molecular marker of cancer or other diseases [32, 33]. With the deepening of the study of AL, the relationship between miRNA and other epigenetic regulation has become a hot spot in tumor research, and the relationship between miRNA and pathological genes closely related to the development and prognosis of AL has been further clarified [34]. miR-126-5p was found to be expressed in blood cells, such as granulocytes, monocytes, and leukemia cells, through bioinformatics methods, which can promote leukemic cells to differentiate and mature into granulocytes [35]. One of the important mechanisms of retinoic acid in inducing leukemia differentiation is to induce the expression of miR-126-5p. miR-13 was found to be upregulated in chronic kidney disease patients [36].

In this study, the median OS time of patients with high miR-126-5p expression was significantly higher than that of patients with low miR-126-5p expression, and the median OS time of patients with high miR-13 expression was significantly higher than that of patients with low miR-13 expression, suggesting that miR-126-5p and miR-13 can be used as potential targets for clinical treatment of acute myeloid leukemia. The expression level of miR-126-5p was significantly correlated with lactate dehydrogenase level, HGB level, NPM1 mutant type, and complete remission (*P* < 0.05), but not with sex, age, WBC, PLT, proportion of bone marrow primordial cells, hepatomegaly, splenomegaly, lymph node enlargement, and FLT3-ITD (*P* > 0.05). The expression level of miR-13 was significantly correlated with HGB level, NPM1 mutant type, and complete remission (*P* < 0.05), but not with sex, age, lactate dehydrogenase level, WBC, PLT, proportion of bone marrow primordial cells, hepatomegaly, splenomegaly, lymphadenomegaly, and FLT3-ITD (*P* > 0.05). Cox multivariate regression analysis showed that peripheral blood WBC, bone marrow blast cell count, and miR-126-5p and miR-13 were prognostic factors in patients with acute promyelocytic leukemia (*P* < 0.05). The relative expressions of serum miR-126-5p and miR-13 have high sensitivity, specificity, accuracy, and AUC in predicting 1-year death in patients with acute promyelocytic leukemia, suggesting that serum miR-126-5p and miR-13 are closely related to the prognosis of acute promyelocytic leukemia. It is further suggested that the levels of serum miR-126-5p and miR-13 can be used as important indicators to evaluate the prognosis of patients with acute promyelocytic leukemia.

To sum up, serum miR-126-5p and miR-13 are closely related to the prognosis of patients with acute promyelocytic leukemia. Serum miR-126-5p and miR-13 can be used as reliable indexes to predict the prognosis of patients with acute promyelocytic leukemia, which can provide reference for improving the therapeutic effect and predicting prognosis of acute promyelocytic leukemia and adjust the treatment strategy in time to strive for more chances of survival for patients.

## Figures and Tables

**Table 1:** Comparison of miR-126-5p and miR-13 expression levels between the two groups.

Group	Number of cases	miR-126-5p expression levels	miR-13 expression levels
Observation group	129	12.31 ± 2.25	16.05 ± 3.47
Control group	74	17.30 ± 3.28	21.66 ± 2.18
*t*		12.814	12.552
*P*		<0.001	<0.001

**Table 2:** comparison of miR-126-5p and miR-13 expression levels in patients with different characteristics.

Clinicopathological features	n	miR-126-5p	*t*	*P*	miR-13	*t*	*P*
Gender							
Male	95	15.51 ± 2.68	0.848	0.396	19.57 ± 2.30	1.066	0.288
Female	108	15.79 ± 2.01			19.90 ± 2.11		
Age (years old)							
<50	120	16.91 ± 1.73	0.279	0.780	18.91 ± 1.73	0.296	0.772
≥50	83	16.84 ± 1.79			18.84 ± 1.63		
Lactate dehydrogenase							
<320	92	15.65 ± 1.29	14.972	<0.001	18.06 ± 1.22	0.584	0.560
≥320	111	17.76 ± 0.67			17.91 ± 2.20		
WBC (×10^9^/L)							
<10	21	15.44 ± 1.16	0.109	0.914	18.39 ± 1.25	0.057	0.955
≥10	182	15.48 ± 1.64			18.37 ± 1.56		
HGB (g/L)							
<90	151	16.06 ± 2.47	6.282	<0.001	18.92 ± 1.25	5.320	<0.001
≥90	52	18.74 ± 3.13			20.21 ± 2.09		
PLT (×10^9^/L)							
<50	149	16.81 ± 1.67	0.815	0.416	19.73 ± 2.54	1.105	0.271
≥50	54	17.05 ± 2.29			20.16 ± 2.18		
Proportion of bone marrow primordial cells							
<0.6	130	15.98 ± 2.01	0.171	0.864	17.67 ± 2.38	1.642	0.102
≥0.6	73	16.03 ± 1.98			18.21 ± 1.99		
Hepatomegaly							
Yes	142	16.28 ± 2.11	0.254	0.800	18.32 ± 2.62	1.119	0.264
No	61	16.36 ± 1.92			17.81 ± 3.68		
Splenomegaly							
Yes	155	17.87 ± 2.50	0.144	0.885	19.29 ± 2.75	1.621	0.107
No	48	17.93 ± 2.57			20.03 ± 2.81		
Lymph node enlargement							
Yes	136	15.37 ± 2.56	0.635	0.526	18.91 ± 2.07	0.400	0.689
No	67	15.61 ± 2.48			19.04 ± 2.38		
NPM1							
Mutant type	127	17.23 ± 2.19	4.872	<0.001	19.03 ± 2.16	6.081	<0.001
Wild type	76	18.96 ± 2.83			20.82 ± 1.79		
FLT3-ITD							
Positive	125	17.95 ± 2.14	0.200	0.842	18.55 ± 2.03	0.664	0.504
Negative	78	18.01 ± 1.99			18.76 ± 2.42		
Complete remission							
Yes	181	19.38 ± 1.86	5.147	<0.001	18.53 ± 2.29	4.620	<0.001
No	22	17.21 ± 1.93			16.17 ± 2.10		

**Table 3:** Cox multivariate regression analysis.

Variable	B	SE	Ward	P	OR
Peripheral blood WBC	1.135	0.417	3.188	0.039	(1.089-8.513)
Bone marrow blast cell count	1.462	0.963	2.614	0.014	(1.218-6.137)
miR-126-5p	1.342	0.792	3.536	<0.001	(1.896-13.180)
miR-13	1.090	0.365	9.736	<0.001	(1.659-12.042)

## Data Availability

The analyzed data sets generated during the study are available from the corresponding author on reasonable request.
